# Multidisciplinary clinician perceptions on utility of a machine learning tool (ALERT) to predict 6‐month mortality and improve end‐of‐life outcomes for advanced cancer patients

**DOI:** 10.1002/cam4.70137

**Published:** 2025-03-03

**Authors:** Nithya Krishnamurthy, Melanie Besculides, Ksenia Gorbenko, Melissa Mazor, Marsha Augustin, Jose Morillo, Marcos Vargas, Cardinale B. Smith

**Affiliations:** ^1^ Internal Medicine Icahn School of Medicine at Mount Sinai New York New York USA; ^2^ Institute for Healthcare Delivery Science Department of Population Health Science and Policy, Icahn School of Medicine at Mount Sinai New York New York USA; ^3^ SUNY Downstate Health Sciences University College of Medicine New York New York USA; ^4^ Division of Hematology, Medical Oncology and Brookdale Department of Geriatrics and Palliative Medicine Icahn School of Medicine at Mount Sinai New York New York USA

**Keywords:** cancer management, clinical management, predictive model, quality of life

## Abstract

**Background:**

There are significant disparities in outcomes at the end‐of‐life (EOL) for minoritized patients with advanced cancer, with most dying without a documented serious illness conversation (SIC). This study aims to assess clinician perceptions of the utility and challenges of implementing a machine learning model (ALERT) to predict 6‐month mortality among patients with advanced solid cancers to prompt timely SIC.

**Methods:**

One‐on‐one semi‐structured interviews were conducted with oncology physicians, advanced practice providers, registered nurses, and social workers until knowledge saturation was reached (*N* = 19). Thematic analysis was conducted on the transcribed interviews, which were reviewed and coded by a team of interdisciplinary investigators.

**Results:**

Clinician‐perceived benefits were (1) guiding prognostication and the objectivity of the prediction easing clinician distress with EOL treatment planning; (2) standardizing prognosis discussions across specialties, limiting aggressive EOL procedures; (3) respecting patient values by providing them time to get affairs in order and plan for cultural EOL rituals; and (4) facilitating earlier SIC and palliative care referrals. Challenges identified were (1) integration of predictions with clinical expertise; (2) balancing the reliability and accuracy of the model with a rapidly evolving therapeutic landscape; and (3) concern about patient distress due to poor communication.

**Conclusions:**

Clinicians expressed widespread acceptability of ALERT and identified clear benefits, particularly in triggering earlier SIC and standardizing prognosis discussions across care teams to avoid aggressive hospital interventions at EOL. Challenges identified, including concerns regarding communication of the prediction and integration with clinical expertise and new research, will guide refinement of the ALERT model.

## 1 INTRODUCTION

Studies consistently show historically minoritized patients with advanced cancers have infrequent and inadequate goals of care (GoC) conversation with their oncologists compared to their white counterparts.^1^, ^2^, ^3^ A GoC conversation involves a discussion between a healthcare clinician and a patient or their family to understand the patient's values, preferences, and medical care goals within the context of their prognosis and available treatments.^4^ Adequate GoC conversations help the care team plan treatments based on the patient's stated goals and avoid unnecessary end‐of‐life (EOL) treatments.^5^, ^6^ Despite these benefits, most minoritized patients die without having a documented conversation, commonly referred to as serious illness conversation (SIC) with their care team.^7^ Most clinicians only initiate SICs with their patients late in the trajectory of cancer, often in the patient's last month of life.^8^ One of the obstacles to early SIC is a lack of validated prognostic tools to predict short‐term mortality in patients with advanced cancer. Prognostic uncertainty coupled with racial bias and structural racism may contribute to communication barriers, with clinicians assuming that individuals from minoritized backgrounds may not want to engage in these crucial conversations, may have lower health literacy or express pain or emotions differently.^9^, ^10^

Recent studies of machine learning (ML) utilizing electronic health record data have demonstrated strong potential in oncology, from early cancer detection to prognostication. Utilizing a lightweight deep convolutional neural network (CNN) architecture, with ultrasound (BUSI) and mammography (mini‐DDSM) datasets for training and evaluation significantly enhances the precision and reliability of breast cancer diagnosis, achieving malignancy identification accuracies of 97.50% and 92.31%, respectively.^11^ Similarly, a novel sparse‐based deep cascade blood cancer detection network demonstrated excellent performance on small databases, and a deep CNN for classifying brain tumors on small datasets has shown promising results.^12^, ^13^ ML models have also shown the capability to effectively identify patients at a high‐risk of short‐term mortality.^14^, ^15^, ^16^ Recently, a ML model accurately predicted 30‐day mortality in a large cohort of cancer patients starting chemotherapy.^17^ Another study validated a ML model for predicting 6‐month mortality in advanced cancer patients which can encourage clinicians to consider earlier initiation of SIC.^18^, ^19^ ML models have the potential to overcome obstacles that impede early SIC including addressing current tools' limitations in identifying patients at risk for short‐term mortality and mitigating the impact of structural racism, which may hinder healthcare providers from initiating SIC in a timely manner.^20^, ^21^ However, there remains a lack of research assessing multidisciplinary clinician perspectives regarding integration of predictive models into clinical decision making, which is critical for implementation success.

To our knowledge, our study is the first to assess multidisciplinary, including social work and nursing, perspectives on benefits and challenges of incorporating a ML model (ALERT) to predict 6‐month mortality in patients with advanced cancers. The goal is to incorporate feedback prior to implementation so the model is successful in improving SIC among patients, particularly among those from minoritized backgrounds who face multifactorial barriers to early SIC.

## 2 METHODS

Semi‐structured qualitative interviews were conducted with outpatient multidisciplinary clinicians, including advanced practice providers, social workers, registered nurses, and physicians, who cared for patients with advanced cancers in the ambulatory setting. Clinicians were asked to share their perspectives on concerns, beliefs, and thoughts about using a predictive mortality tool to improve EOL outcomes and increase prognosis discussions and SICs. Interviews were approximately 30 min long and conducted in English via HIPAA‐compliant Zoom. To increase transparency and credibility, results are reported using the consolidated criteria for reporting qualitative research.^22^ This was an institutional review board approved study (STUDY‐22‐01543). Prior to participation, all clinicians provided verbal informed consent.

### 2.1 Participants and setting

Multidisciplinary clinicians who care for advanced cancer patients were recruited from urban ambulatory care centers via purposive sampling. Clinicians were selected to represent a variety of specialties, including breast, gastrointestinal, neurological, blood, dermatological, and head and neck. The sample was also designed to capture variation across the different roles people play in utilizing mortality data to inform SIC. Stakeholders were interviewed until knowledge saturation was reached (*N* = 19).

### 2.2 Data collection

Interviews were conducted using a semi‐structured interview guide (Table S1) that was designed to encourage in‐depth discussion of benefits and challenges of the predictive model. The first two interviews were conducted jointly to ensure the questions were understood and elicited the information of interest; the guide was then slightly revised. Interviews were recorded with permission, and professionally transcribed verbatim.

### 2.3 Data analysis

Transcripts were coded and analyzed using Dedoose qualitative analysis software. An inductive thematic analysis approach was employed.^23^ A seven‐person coding team consisted of a qualitative researcher (K.G.), a nurse and palliative care researcher (M.M.), trained student research interns (N.K., M.V., L.G.), and clinical research coordinators (M.A., J.M.), Each interview was read and open coded by team members. The team then convened to review their individual coding and reach agreement regarding any discrepancies. After each interview, the emergent codebook was applied to the next interview. All transcripts were then re‐coded by two coders per transcript using the finalized codebook. Codes pertaining to benefits and challenges of implementing the ML model were collated into descriptive themes (N.K.). All themes and subthemes were reviewed by all authors, including the PI (C.S.) and a qualitative research expert (M.B.), to ensure rigor of analysis and consistency of interpretation (Table S2).

### 2.4 Clinician characteristics

Nineteen interviews were conducted with multidisciplinary clinicians. As detailed in Table 1, clinician participants were in practice for an average of 15 years (range 2–42). Demographic information was available for 18 clinicians, of whom 72% identified as female. 67% of participants self‐identified as White, 7% as Black, and 27% as Asian. Clinicians from various cancer specialties and with diverse roles participated in the study (*N* = 18 with complete information). There was an even distribution of oncologists, social workers and nurse practitioners (28%), with registered nurses comprising 11% of the group.

**TABLE 1:** Clinician characteristics.

Clinician characteristics (*N* = 19)^a^	
Years in practice	15 (2–42)
Sex, % (*N*)	
Female	72% (13)
Male	28% (5)
Race, % (*N*)	
White	67% (12)
Black	7% (1)
Asian	27% (5)
Cancer specialty (*N* = 18), % (*N*)	
Breast	11% (2)
Neurological	11% (2)
Head and neck	17% (3)
Blood	11% (2)
Gastrointestinal	22% (4)
Thoracic	11% (2)
Dermatologic	11% (2)
Unknown	5.6% (1)
Clinician role, % (*N*)	
Oncologist	27.8% (5)
Social worker	27.8% (5)
Nurse practitioner	27.8% (5)
Registered nurse	11.1% (2)

*Note*: Clinician demographic, practice, and clinical characteristics.

^a^ Data were only available for 18/19 clinicians.

### 2.5 Qualitative findings

Qualitative analysis of interview transcripts from multidisciplinary clinicians identified four benefits and three challenges of implementing a ML predictive model in EOL care planning for advanced cancer patients (Table 2). A detailed discussion of their perspectives follows below.

**TABLE 2:** Interview guide example questions^a^.

Theme	Example questions
Goals of care conversations	For patients who have advanced cancer, can you describe your conversations about goals of care?
Timing and format of prognosis discussions	When do you tend to discuss prognosis with your patients and their family? How do you do that? What might help you in these conversations? I understand there are many challenging aspects about prognosis conversations. What type of information or support could be helpful in making those conversations easier?
Integration of prediction into clinical workflow	If you were able to receive predictions about the probability of your patients' 6‐month mortality, how would you handle this information? When would be a good time to see this information?
Application and implementation of ML predictive model	Would you share it with patients/family? How? When would you not share it? How would you like that information to be shared with you? What are some of the foreseeable benefits/challenges with integrating this tool? What are some key aspects we should be considering with the integration of the tool?

*Note*: Sample questions from semi‐structured qualitative interviews exploring perspectives on concerns, beliefs, and opinions on a predictive mortality tool to improve end‐of‐life outcomes and increase SICs.

^a^ The full interview guide is presented in Table S1.

#### 2.5.1 Theme 1: Guiding prognostication and easing clinician distress with EOL decision making

A major benefit noted by clinicians was the value of the ML prediction in guiding prognostication, particularly in areas of uncertainty such as newly diagnosed patients with metastatic disease. A GI oncologist explained the challenges in initiating discussions surrounding EOL care for these patients: *“My personal weakness is on newly diagnosed patients*. *These are quite often the inpatients that we see*. *They're still*, *despite a lot of experience—personal experience as well as familiarity with literature—that trying to resurrect them*, *trying to start chemotherapy and finesse our ways out of the hospital and into the ambulatory setting*. *Wow*, *getting a machine‐based patient‐specific data on this to discuss patients*, *to talk about why not offering chemotherapy is reasonable with patients and families*.*”* Oncologists underscored that having data from a ML model would be valuable in terms of encouraging treatment planning discussions with patients and families and could help mitigate overtreatment. An inpatient social worker provided additional perspective on how the ML tool could ease clinician distress with EOL decision making given the strong nature of the clinician–patient relationship, explaining the prediction may *“temper some oncologists' optimism on how long their patients have… And I see this often*, *there will be oncologists who develop really*, *really strong relationships with their patients*. *It will be a patient they love*, *and I know that they have a lot more challenging time telling those patients*, *I'm so sorry I can't give you any more treatment*, *because they're humans*. *They're emotionally invested*.*”*

#### 2.5.2 Theme 2: Standardizing prognosis discussions across the care team

Clinicians identified the potential of the ML model to standardize prognosis communication across interdisciplinary teams, to reduce patient distress and aggressive procedures at EOL. For example, an oncology nurse practitioner hoped the tool would alert clinicians to check in with the oncology team to ensure the planned procedure or medication change matched the patient's GoC. The clinician described having “*a patient who had a vascular issue*, *he had osteomyelitis, and he had a very limited prognosis from his cancer*. *The vascular surgeon wanted to take him for an amputation*. *It's like*, *is that really in line with his goals of care right now when he knows? And so I think it might be helpful for clinician to clinician, if it was presented in Epic*, *like a BPA type thing*. *Like this patient has a predicted mortality*. *And that clinician can choose to just dismiss it*, *like this doesn't involve me*. *Or it might ring into them to be like*, *maybe I should reach out to the oncologist before I move forward with this procedure*, *or this medication or something like that*.*”* Likewise, an outpatient social worker emphasized the importance of consistent and clear discussions surrounding prognosis, urging all members of the care team to *“share the same message—because there are so many different members of a team*. *You've got an NP*, *or an RN*, *and you've got social work*, *and you've got a dietician*. *Everyone needs to be saying the same thing*.*”*

#### 2.5.3 Theme 3: Incorporating patient values at EOL

Practitioners recognized that the ML prediction could increase incorporation and respect for patient values at EOL, affording individuals the opportunity to get affairs in order, spend meaningful time with family, and plan for cultural EOL rituals. For example, a social worker explained “*I think especially our Chinese immigrant patients*, *a lot of them from this cultural perspective*, *I can tell that a lot of them want a certain number*. *They would like to ask how much time I have… The funeral is so important to them*, *so for some of them it's so important for them to prepare everything properly*. *I have so many things to prepare for before I die*. *Like I have so many relatives I need to talk to*, *if I know this is the exact time period I have*, *I can plan accordingly so that I make sure I achieve as much as possible*, *what I want to do*.*”* Clinicians noted the anxiety patients grapple with due to the variable trajectory of cancer, acknowledging that the prediction could provide a semblance of certainty to help elucidate EOL goals. A neurooncology nurse practitioner emphasized encouraging patients to fulfill personal desires, for example to *“go visit somebody that you haven't seen for ten years, stuff like that*,*”* with the goal of “[*giving*] *them valuable time*. *I think that would be a great benefit*, *if we could give some accurate information about how long they can live*.*”*

#### 2.5.4 Theme 4: Nudging earlier SIC and palliative care (PC) referrals

Clinicians anticipated that the ML prediction would nudge practitioners to engage in earlier SIC and referrals to PC. Participants explained that having a ML‐derived prognosis to discuss with patients and their families could help in realistically weighing the symptoms and side effects versus benefits of additional treatment, to better preserve quality of life. A social worker explored how the prediction could help families and patients engage with PC earlier and prevent a protracted EOL hospital course, stating “*We'll give eight rounds of treatment*, *even if they haven't woken up*. *And I think a part of that is because the families do think—because they guess this is possible—that they will recover*. *But I think if we had a way to know that they wouldn't*, *I think we would be able to transition sooner and it would be much more peaceful*. *Oftentimes*, *that patient will die in the hospital*. *And I rarely am able to get them home*, *because they're in so much pain that they're needing IV meds*, *so home isn't an option*.*”* A GI oncologist shed light on how the prediction could factor into individualized treatment planning and determining when to transition care, explaining “*It's sometimes kind of a reality check for the physician to say*, *you know this predictive tool does say that the patient is sicker that we expect*, *than you think*. *It does help to trigger more consideration about what the patient could be*.*”* Similarly, an advanced practice provider noted that “*we might be apt to make the referral to supportive care sooner*, *if I had that information*.*”*

#### 2.5.5 Theme 5: Integration of predictions with clinical expertise and experience

Clinicians cautioned against overreliance on the prediction provided by the ML model. Emphasizing the nuances of patient care and the limitations of the tool, participants urged integration of clinician expertise and experience in interpretation of the ML prediction and in treatment planning. A nurse practitioner explained *“So I think as a clinician*, *you just get the numbers from it*, *which is straightforward*. *That's probably all we expect from the machines*, *or artificial intelligence. Then the rest*, *I think we should input our judgment*, *our clinical data*, *we should interpret other aspects*, *and then process it and then make the recommendation*.*”* An oncology nurse expanded on the importance of individualized treatment planning, describing “*I've been a nurse for 26 years…Everything is very individualized*, *and each patient's experience is completely different from the next*, *and their outcomes are completely different*.*”* Practitioners underscored the importance of communicating limitations of the ML prediction when discussing prognosis with patients. One clinician envisioned how the information could be presented to patients, explaining “*Is the statistic going to work to our detriment if we say this is the statistic*, *and someone doesn't even live as long as predicted? How does that impact our situation? I tend to always say*, *there's populations outside the statistic oftentimes*, *and we just can't predict who those people will be*.*”*

#### 2.5.6 Theme 6: Balancing the accuracy of the model with incorporation of an evolving therapeutic landscape

Clinicians called for transparency surrounding the tools' validation and explained that they would want to have information about accuracy, reliability, and data inputs and development of the ML model before incorporating the predictions into prognostication and sharing the results with patients. One oncologist mentioned *“I think I'd want to see data on its actual accuracy before I start modifying any decisions based on that*. *I think that this is something that will take time for people to be able to accept*.*”* Clinicians also wanted to understand how the ML model would integrate rapidly evolving therapies and trials in oncology that may alter prognostication. An oncologist expanded on concerns about the tool's ability to adapt to the evolving therapeutic landscape, stating *“I think it's also important to say that this is what it is right now*, *from what we know right now*. *From the data that we have right now*, *this is the prognosis…Therapies change*, *trials become available*. *We're always learning more*, *which may alter this prediction*.*”*

#### 2.5.7 Theme 7: Concern about patient distress and emotional reaction to prediction

Practitioners expressed concern about patients' reaction to the prediction; they explained that patients are at different stages of acceptance of their prognosis, and the prediction may cause significant stress or depression to those who may not want to face a stark reminder of their poor prognosis. Nuanced and empathetic communication of the prediction was critical, in a way that maintained hope while offering the patient a realistic prognosis. One oncologist elaborated “*You know*, *challenge number one is you never want to take away hope*. *Especially when people have advanced cancer*. *It's getting to know somebody quickly*, *and the kind of person they are*. *…*. *The hardest part is finding a way to deliver bad news in a delicate way*, *while also trying to maintain hope*.*”* Another oncologist expressed concerns that the prediction may disrupt the natural process of recognition of physical decline at the end stages of advanced cancer, explaining, “*We want to think about the potential therapeutic benefit of that, versus the potential toxicity of that signal*. *That's one thing*. *Patients know when they begin to deteriorate*. *Patients with cancer*, *many other diseases*. *It's gentle*, *it's natural*. *It's fatigue that's both a physical fatigue—we get them out of pain*, *we get them out of breathlessness*. *We have interventions for that*. *And their fatigue is not just physical*, *but I think there's a bit of an emotional fatigue that really often manifests as peace*, *peacefulness*. *I don't want to disturb the peace*.*”*

## 3 DISCUSSION

Our study found that clinicians expressed widespread acceptability of ALERT and identified clear benefits, particularly in triggering earlier SIC and standardizing prognosis discussions across care teams. Clinicians expressed concerns about accuracy and patient reactions to the prediction, but felt that the ML tool had the potential to facilitate earlier SIC. This sentiment is supported by data indicating that ML tools can enable earlier SIC and palliative care consults. A recent study found that ML prognostic models decreased use of aggressive chemotherapy at EOL and increased SIC frequency fourfold. However, overall frequency remained low and SIC rates were not sustained over time, suggesting further assessment of implementation barriers is necessary.^24^

Research also indicates that there are many missed opportunities for SIC in oncology patients which could potentially be mitigated by a tool that nudges clinicians to initiate SIC. A sample of 141 patients and 39 oncologists indicated that out of 423 patient–clinician encounters, only 21 included EOL care discussions.^25^

Another study revealed that SIC rates remained below 15% for high‐risk patient groups, even with ML‐driven behavioral nudges.^26^ Our multidisciplinary perspectives provide additional insight into the barriers clinicians face to initiating SIC. Developing strong interpersonal relationships with patients makes it harder to shed optimism about their prognosis. Furthermore, clinicians frequently encounter difficulties in timing SIC, as they navigate the intersection of medical evidence, emotions, and patient preferences. Our research suggests that clinicians perceive ALERT, with its objective survival prognostication, as a viable solution to these challenges.

Our study identified that a key benefit of the ALERT model was that the objectivity of the prediction could ease clinician distress with EOL treatment planning. Clinician moral distress with EOL care discussions is well recognized as a cause of burnout in oncology care teams. A qualitative study found that there was a connection between burnout and clinician‐perceived ineffective communication strategies with patients who were dying.^27^ A personal sense of failure during EOL treatment planning has also been described as a frequent cause of burnout in oncology care teams and could affect initiation of SIC, and providing clinicians with prognosis assistance could help mitigate burnout.^28^

Potential challenges to implementing ALERT identified by multidisciplinary oncology care clinicians included concerns about the predictive accuracy of the ML model and its ability to keep up with rapidly evolving clinical trial data. Concern about factors such as automation bias, where clinicians may over rely on the predictive capabilities of the ML tool, played a role in clinician hesitation to adopt a tool like ALERT.^29^ To our knowledge, studies on the impact of new and targeted treatments in oncology and predictive accuracy of ML models have not been published, but educating clinicians on the variables determined to be important for prognostication has the potential to increase trust in the predictive model. A recent cohort study of 43, 274 patients demonstrated that the ALERT model had reasonable concordance in model discriminatory performance and fairness metrics (equal opportunity, equalized odds, and disparate impact) among racial subgroups, showing promise for application in clinical settings to improve SIC frequency for minoritized groups.^30^

Patient distress and emotional reactions to prognostic prediction was a concern of many clinicians. Prior research supports our findings; oncology clinicians often report inadequate training to have these complex conversations and a frequently cited barrier to initiating prognosis discussions is oncologists' fear that disclosing prognostic information would make patients feel hopeless or depressed, and hinder the patient–physician relationship.^31^ However, studies have shown that prognostic disclosure was associated with more realistic expectations and EOL treatment choices more in line with patient preferences, without negatively impacting survival, sense of hope, emotional well‐being, or the patient–physician relationship.^32^, ^33^, ^34^ Some patients may not be ready for or may prefer not to explicitly discuss life expectancy,^35^ and communication should be tailored to patient and caregiver preferences, with consideration of whether prognostic discordance hinders goal‐concordant care. Future directions of this work include studying patient perspectives on the ML mortality prediction, receptiveness to direct prognostic information, output of the prediction, and communication preferences. Incorporation of both clinician and patient perspectives will be critical in the eventual implementation of ALERT into clinical workflows to nudge earlier SIC and address disparities in SIC frequency among minoritized groups.

Our study has a few limitations. First, the findings are limited by sampling from an urban research institution and our participants identified as majority non‐Hispanic white (67%) and female (80%), and thus our results may not generalize to clinician perspectives from different demographic backgrounds or rural practice settings. However, the racial and ethnic demographics of clinicians as well as the sex of the APP, nursing and social work groups in our sample are consistent with the oncology workforce nationwide. Further research could ascertain palliative care physicians' experiences on ML‐driven SIC and referrals to supportive care, as well as include multiple care settings.

## 4 CONCLUSION

Our study found that clinicians expressed widespread acceptability of ALERT and identified clear benefits, particularly in triggering earlier GoC conversations and standardizing prognosis discussions across care teams with the goal of avoiding aggressive hospital interventions at the end‐of‐life. Challenges identified, including concerns about how to best communicate the prediction output and integration of the prediction with clinical expertise and new research, will guide ongoing development and eventual implementation of the ALERT ML predictive model. Future research will explore patient perspectives on the ML model and preferences for predictive output and integration into SIC, to guide further refinement of ALERT.

## AUTHOR CONTRIBUTIONS

**Nithya Krishnamurthy:** Data curation (equal); formal analysis (lead); investigation (lead); methodology (equal); software (equal); validation (equal); visualization (equal); writing – original draft (lead). **Melanie Besculides:** Data curation (equal); investigation (equal); supervision (equal); validation (equal); writing – review and editing (equal). **Ksenia Gorbenko:** Data curation (equal); investigation (equal); supervision (equal); writing – review and editing (equal). **Melissa Mazor:** Supervision (equal); validation (equal); writing – review and editing (equal). **Marsha Augustin:** Data curation (equal); investigation (equal); methodology (equal). **Jose Morillo:** Data curation (equal); investigation (equal); methodology (equal); project administration (equal). **Marcos Vargas:** Data curation (supporting); investigation (equal); methodology (equal). **Cardinale B. Smith:** Conceptualization (lead); funding acquisition (lead); project administration (equal); resources (equal); supervision (equal); validation (equal); writing – review and editing (equal).

## CONFLICT OF INTEREST STATEMENT

The authors declare no conflicts of interest.

## ETHICS STATEMENT

This project was approved by Mount Sinai institutional review board (IRB) (STUDY‐22‐01543).

## PRECIS

Clinicians expressed widespread acceptability of ALERT for its potential to facilitate early serious illness conversations and standardize prognosis discussions. However, challenges remain in effectively integrating predictions with clinical expertise and adapting to evolving therapies.

## Supporting information


Data S1.

## Data Availability

Access to the data supporting the findings of this study is available upon request from the corresponding author. The data is not publicly available due to privacy or ethical considerations. The qualitative data analyzed in this study are available upon request from the corresponding author, ensuring confidentiality and ethical considerations.
