# Supplementary material for: Multidisciplinary clinician perceptions on utility of a machine learning tool (ALERT) to predict 6‐month mortality and improve end‐of‐life outcomes for advanced cancer patients
Source: Cancer Med. 2025 Mar 3;14(5):e70137. doi: 10.1002/cam4.70137 (PMC11875110; doi:10.1002/cam4.70137)
Supplement: Supplementary file 1 — Data S1. [file CAM4-14-e70137-s001.docx]

**Supplemental Table 1. Clinician Interview Guide**

Semi-structured Interview Guide (Clinician)

Introduction for clinician participants:

Thank you for taking the time to speak with me today. We are talking to oncology clinicians who see patients with solid tumors in the ambulatory setting. We will speak to about 10 oncologists in this project.

I would like to record this interview if you don’t mind so we can capture your words exactly. When I start the recording, I will try to avoid using your name so your information remains confidential. If I do use your name or if you mention any identifying information, we will remove it from the transcript so the data cannot be traced directly to you. Feel free to skip any questions or ask me to pause the recording if you would like to say something off the record. Do you have any questions before we begin? Is it okay for me to start the recording?

<Start recording>

Topics and sample questions (to be further tweaked before the beginning of patient and physician recruitment)

1. Clinical practice: Can you tell me a little bit about your practice (inpatient, outpatient, hospice?) Do you often diagnose patients with cancer, or do many of your patients come with a diagnosis already?
2. Decision making: How do you make decisions about treatment? How do you see your role and your patient’s role?
3. Goals of Care: For patients who have advanced cancer, can you describe your conversations about goals of care? Follow up with questions about challenges and helpful aspects.
4. Prognosis and prognostication: When do you tend to discuss prognosis with your patients and their family? How do you do that? What might help you in these conversations?
5. Ideal conversation: I understand there are many challenging aspects about prognosis conversations. What type of information or support could be helpful in making those conversations easier?
6. ML/AI tool: If you were able to receive predictions about the probability of your patients’ six-month mortality, how would you handle this information? When would be a good time to see this information? Would you share it with patients/ family? How? When would you not share it? How would you like that information to be shared with you? What are some of the foreseeable benefits/challenges with integrating this tool?
7. Insight: What are some key aspects we should be considering with the integration of the tool? (e.g., patient education, asking the patient if they want this, communication support, etc)
8. Is there anything else that we didn’t discuss that you’d like to share

Supplementary Table 2: Clinician Codebook

| **Code (Major Categories)** | **Code Definition** | **Examples** |
| --- | --- | --- |
| Practice Setting | The location or clinical setting where the clinician provides cancer care. | Outpatient setting; inpatient setting |
| Patient Population | Includes patients with different cancers, staging, demographics, | Staging of cancer, demographic information |
| Cancer Specialty | The types of cancer that the clinician treats | Breast cancer; lung malignancies; GI cancers |
| Other Services and Referrals | Clinician refers patient to other types of care to help manage disease burden, cancer-related stress, support groups, and other resources; it excludes seeking a second opinion | Hospice care; Palliative Care; social worker; chaplain; pain management doctor; support group; psych consult; stress management; physical therapy |
| Clinician’s Role in Diagnosis | Clinician is part of the cancer diagnosis process | Patient is diagnosed with cancer at urban care center, or at another health system/cancer center |
| Treatment Planning | Clinician’s style or approach to tell the patient what diagnosis they have, and what is known and not known to plan care and treatment options | Explanation of disease, knowledge gaps, staging, and tumor characteristics. |
| Individualized Plan | Tailoring treatment based on patient’s physical and psychosocial status | Considering patient’s comorbidities to adjust treatment plan |
| Active vs. Passive Patient Role | Patient preference to make medical decisions from diagnosis to treatments; it includes Shared Decision-making Preference, | Shared decision-making by clinician and patient, versus if the patient prefers that the clinician decide. |
| Patient Autonomy | Patient’s ability to make their own choices. | Patient is empowered by clinician to make decisions, share thoughts and feelings, etc. |
| Caregiver and Social Support | Patient presenting with family members or friends during visit who provide any form of support. | Caregiver asking questions during a patient visit. |
| Maintaining Hope | Balance of Hope and Reality; Out of Treatment Options; Preparing for bad news | Clinician discusses progression of disease and discusses treatment options and patient preferences moving forward.  Clinician gauging how to balance goals of care conversation with hope/maintaining hope but also with reality which may include delivering bad news, preparing for bad news, how to inform patients that there are no more treatment options |
| Patient Education | Clinician using techniques to ensure patient and family understand diagnosis, treatment, plan | Social Worker using the teach-back method  Ensuring Comprehension |
